# Crohn's Disease Diagnosed in a Man with Sarcoidosis: Coincidence or Correspondence?

**DOI:** 10.1155/2022/5943468

**Published:** 2022-05-27

**Authors:** Krystal Mills, Shaheen Fatima, Norberto Fas

**Affiliations:** Internal Medicine Program, Morehouse School of Medicine, 720 Westview Drive SW, Atlanta, GA 30310, USA

## Abstract

Crohn's disease and sarcoidosis are characterized by noncaseating granulomas, but rarely do they present in the same patient. Their coexistence presents a diagnostic challenge as they are often classified as clinically separate, despite their similarities. We present a case of a 59-year-old man previously diagnosed with pulmonary sarcoidosis who presented to the emergency room with abdominal pain and diarrhea. Colonoscopy revealed multiple ulcers in the colon, with histology in keeping with newly diagnosed Crohn's colitis. The patient had a good clinical response to initiation of steroid therapy and a tumor necrosis factor (TNF) inhibitor.

## 1. Background

Crohn's disease (CD) and sarcoidosis (SA) are both granulomatous disorders, but it is estimated that the concurrence of these diseases in the same patient may be as rare as one person in 10,000,000 [1, 2]. CD is characterized by gastrointestinal involvement [3], whereas SA is characterized by respiratory involvement [4]. However, there are overlapping clinicopathological features which support a possible correlation between these granulomatous disorders.

## 2. Case Report

A 59-year-old African American man with a medical history of pulmonary sarcoidosis presented to the emergency room with a one-month history of abdominal pain and diarrhea. He had been discharged four days prior to this index presentation, after presenting with similar symptoms that was treated as infectious colitis. The abdominal pain was greatest in the periumbilical region and cramping in character. Since the initial onset, the pain changed from intermittent to constant in duration and increased in severity from 4 out of 10 to 10 out of 10. He endorsed associated nausea, unintentional weight loss, bloating, loud bowel sounds, seven to ten loose stools per day, hematochezia, and tenesmus. He also reported a painful genital ulcer and endorsed pain in the distal interphalangeal joints of the fourth digits of his hands that was worse in the morning. He denied other skin changes, fever, chills, vomiting, exposure to ill contacts, new foods, or recent travel. He was a former smoker with a 39-year pack history, drank beer daily, but denied illicit drug use. He denied any personal or family history of inflammatory bowel disease or colon cancer. His colonoscopy three years prior was significant for diverticula and a benign polyp which was removed.

**Figure 1:**
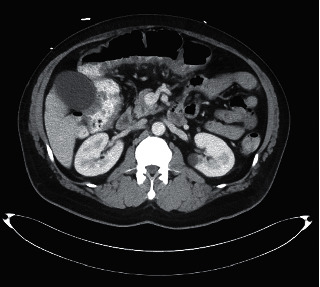
CT abdomen image showing asymmetric wall thickening with subtle adjacent pericolonic fat stranding and mesenteric vessel engorgement involving the transverse and proximal sigmoid colon.

**Figure 2:**
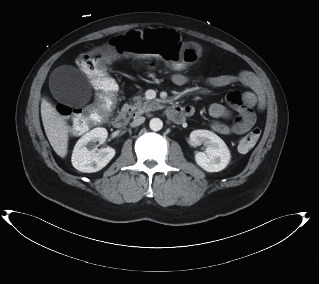
CT abdomen image showing multiple shotty mesenteric lymph nodes, which are nonspecific, possibly reactive related to the colonic process.

On examination, he was in painful distress and tachycardic with other vitals within normal limits. Abdominal examination revealed hyperactive bowel sounds and diffuse abdominal tenderness with voluntary and involuntary guarding. He had a right inguinal genital ulcer and scattered pustules on his back. Laboratory results revealed hemoglobin, 9.2 g/dL; hematocrit, 31.1%; MCV, 76.4 fL; platelet count, 503 K/cmm; ferritin, 13 L; total iron, 13 L; iron saturation, 4; PT, 16.5 sec; INR, 1.35; vitamin K, <50 mcg; albumin, 3.3 g/dL; C-reactive protein, 81.2 mg/L; ESR, >100 mm/hr; and negative tuberculosis skin test. CT abdomen showed asymmetric wall thickening with subtle adjacent pericolic fat stranding and mesenteric vessel engorgement involving the transverse and proximal sigmoid colon (Figures 1 and 2).

**Figure 3:**
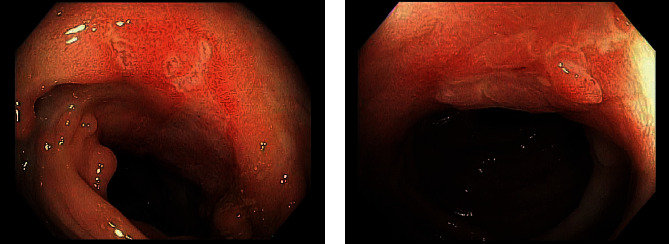
Elective colonoscopy repeated as outpatient was significant for multiple scattered pseudopolyps and discrete ulcerations seen from the transverse colon to sigmoid colon consistent with known Crohn's disease.

Stool studies were negative for infection. Punch biopsy of the genital ulcer done for H&E and infectious studies revealed fibrosis and inflammatory infiltrate, and tissue culture revealed no yeast or fungi. Colonoscopy revealed a large deep ulcer in the midtransverse colon and a few ulcers in the distal descending and sigmoid colon. Colon biopsies resulted as focal active colitis in the right colon, moderate active colitis with crypt abscess formation and ulceration in the transverse colon, and fragments of regenerative/hyperplastic colonic mucosa with ulceration in the descending colon. He was started on solumedrol 30 mg IV twice daily and then transitioned to oral steroids and anti-TNF-alpha therapy. The patient reported improvement in symptoms at one-month follow-up. He had a repeat colonoscopy eight months later that showed persistent active colitis that was improving, and he was continued on anti-TNF-alpha therapy (Figure 3).

## 3. Discussion

Although CD and SA are both granulomatous disorders, their coexistence in the same patient is exceptionally rare [3] and scarcely reported in the literature [5–8]. Noncaseating granulomas are considered the pathologic hallmark of CD and SA [9, 10], but the primary location of granulomas differs. The diseases sometimes mimic each other as respiratory involvement has been reported in up to 40–50% of inflammatory bowel disease (IBD) [4], and gastrointestinal involvement may occur in 10% of sarcoidosis [5].

SA of the digestive tract primarily involves the stomach, unlike the ileocolic involvement seen in CD. Respiratory involvement in IBD varies from airway disease to diseases of the lung parenchymal vessels or pleura and adverse drug reactions [2–4]. Extra-intestinal manifestations of CD parallel the systemic manifestations of SA. In this index case, the patient's groin ulcer was in keeping with pyoderma gangrenosum (PG), which is associated with CD and SA. PG occurs in only 0.5–5% of patients with IBD [11] and exhibits a female preponderance. It is even rarer in patients with SA and typically occurs after trauma. It is unclear whether PG is related to the disease activity of CD or SA, but the use of immunosuppressive agents, which this patient received, resolves the condition.

CD and SA are more common in women and exhibit a peak in the second to third decade of life [12, 13]. The patient in our case was male and older than 40 years, further underscoring his exceptionally unique presentation. The patient was African American, and this race is disproportionately affected by SA [13]. This differs from CD, which is most common in non-Hispanic whites. His history of smoking was his most likely environmental risk factor for CD. His age at diagnosis of CD conferred a good prognosis as persons diagnosed after 60 years of age are less likely to develop complicated CD [12]. There were no granulomas seen on pathology, but they are found in only 30% of patients with CD, more commonly in women and often with more severe disease [14].

At the genetic level, the nucleotide-binding oligomerization domain-containing protein 2 (NOD2) polymorphisms present in CD have been found in a juvenile form of SA known as Blau syndrome [15, 16]. A genome-wide association study performed on CD and SA patients revealed that the most significant association was with the single-nucleotide polymorphism (SNP) rs1398024 on chromosome 10p12.2 and unidentified variants in the C10ORF67 gene region that may be underlying risk factors [17]. Other genetic associations between SA and CD include butyrophilin-like 2 (BTNL2), interleukin-23 receptor, and HERC2 [18].

Theories for coexistence include SA arising as a direct or indirect side effect of the medication used to treat CD. However, in our case, SA preceded CD. In patients with this temporal relation, there are theories that the conditions may be phenotypical manifestations of the same disease spectrum or arise from separate stimuli in which the inciting agent is inhaled in SA and ingested in CD [19]. This case serves to highlight similarities between CD and SA that are not well understood, underscoring the need for further research to identify whether they should remain clinically separate. Although their coexistence is exceptionally rare, new onset CD should be considered in patients with SA who develop gastrointestinal symptoms. Additionally, new onset SA should be considered in patients with CD who develop respiratory symptoms.

## Data Availability

The data used to support the findings of this study are included within the article.
